# Supplementary material for: StackingNet: Collective Inference Across Independent AI Foundation Models
Source: Adv Sci (Weinh). 2026 Jul 9:e76488. Online ahead of print. doi: 10.1002/advs.76488 (PMC13348659; doi:10.1002/advs.76488)
Supplement: Supplementary file 1 — Supporting Information: advs76488‐sup‐0001‐SuppMat.pdf. [file ADVS-9999-e76488-s001.pdf]

Supporting Information for StackingNet: Collective Inference across Independent AI Foundation Models

Siyang Li[1†] *Chenhao Liu*[1†] *Dongrui Wu*[1*] *Zhigang Zeng*[1*] *Lieyun Ding*[2*]

[†]*These authors contributed equally to this work.*

[1]School of Artificial Intelligence and Automation, Huazhong University of Science and Technology, Wuhan 430074, China.
[2]School of Civil and Hydraulic Engineering, Huazhong University of Science and Technology, Wuhan 430074, China.
[*]Corresponding authors. Email: drwu@hust.edu.cn, zgzeng@hust.edu.cn, dly@hust.edu.cn

Supporting Information

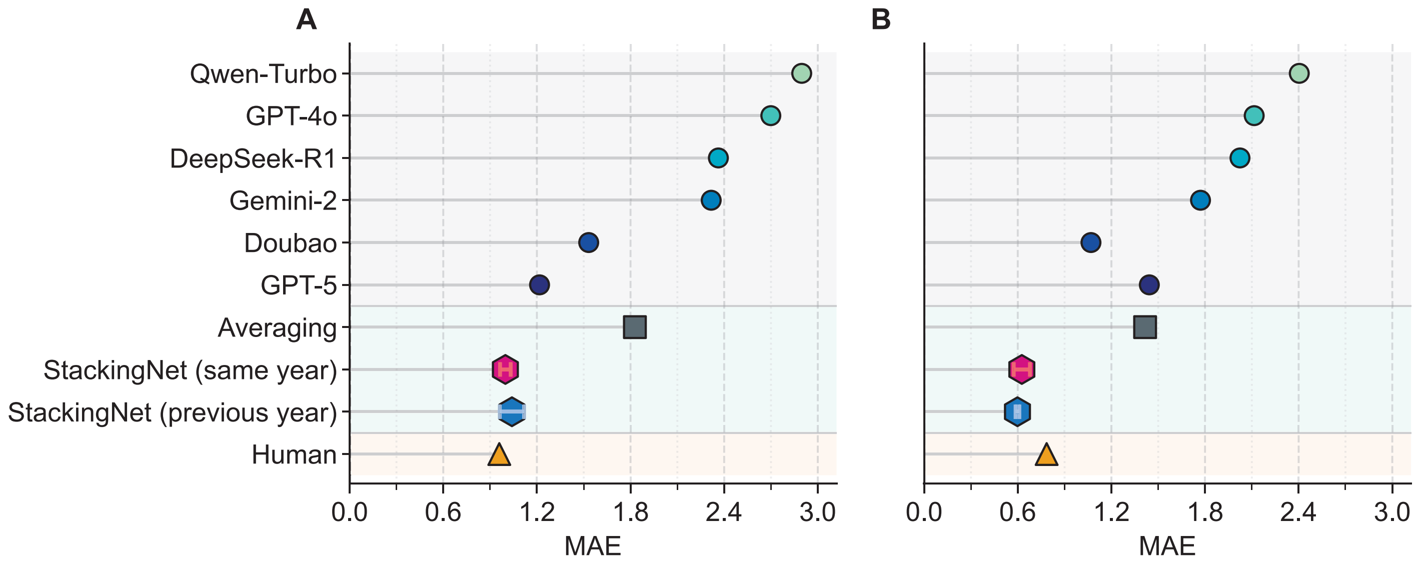

Figure S1: **Research paper rating error by individual human reviewers, individual LLMs, and collective inference of multiple LLMs. a-b,** Mean absolute error (MAE, lower is better) across two datasets, ICLR2025 and NeurIPS2024. StackingNet was trained in a few-shot setting using 1% of labeled examples (10 papers with ground-truth scores from multiple human reviewers) drawn either from the same year or from the previous year.

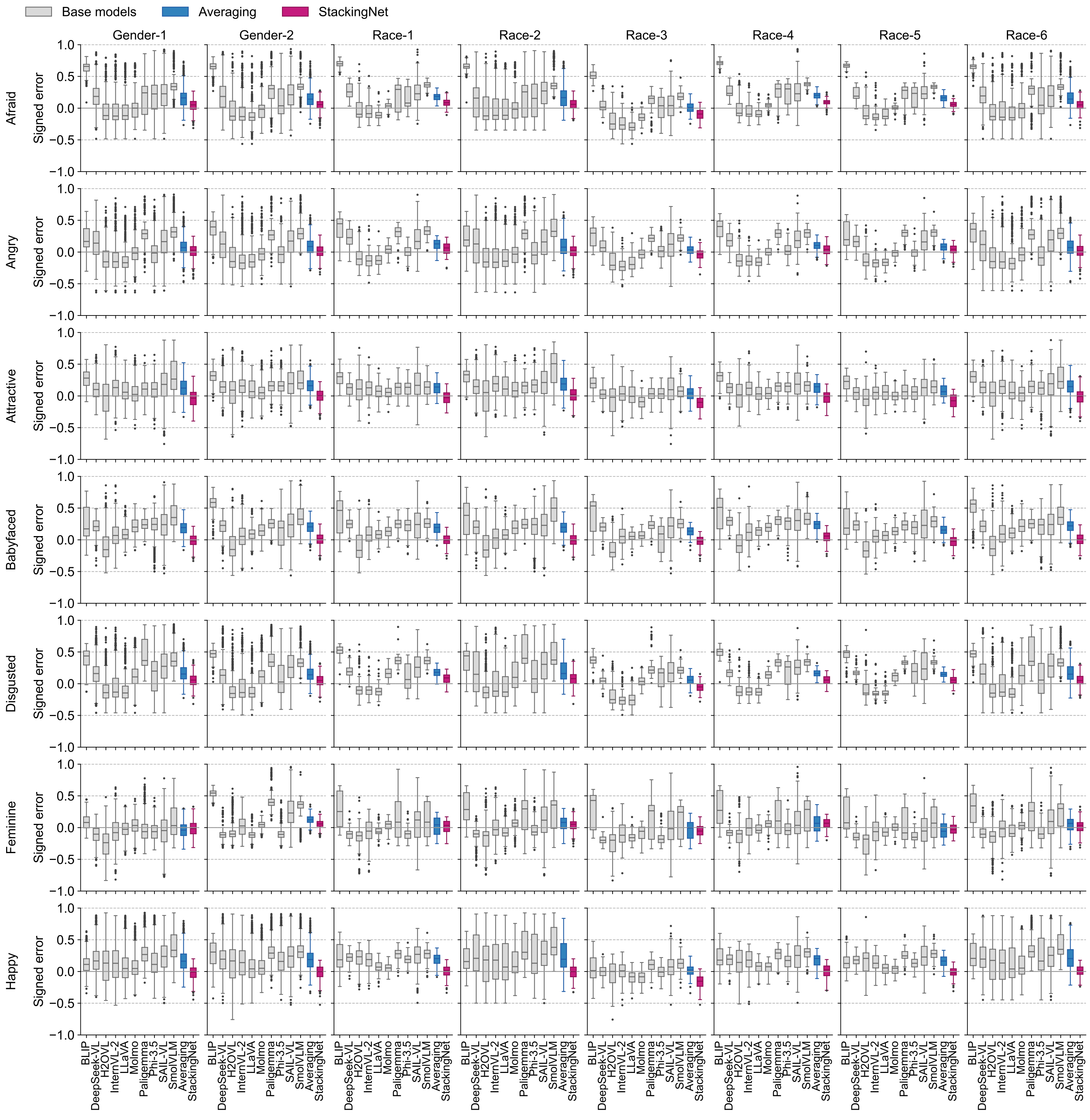

(*Supporting Information Figure 2 continued on next page*)

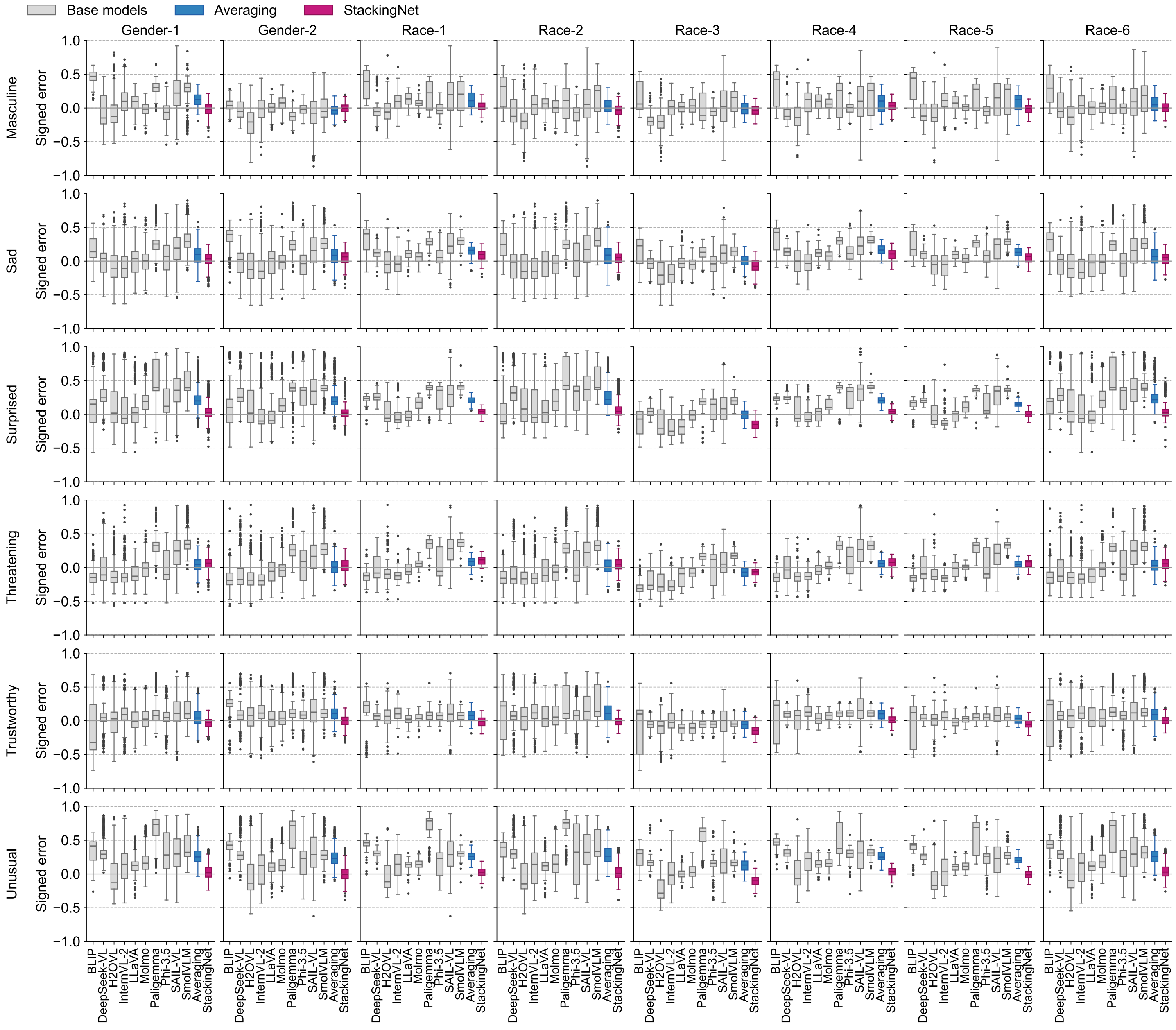

Figure S2: **Facial image attribute ratings by VLMs and StackingNet combination on the Chicago Face Database.** Predicted ratings are shown across gender and race/ethnicity groupings.

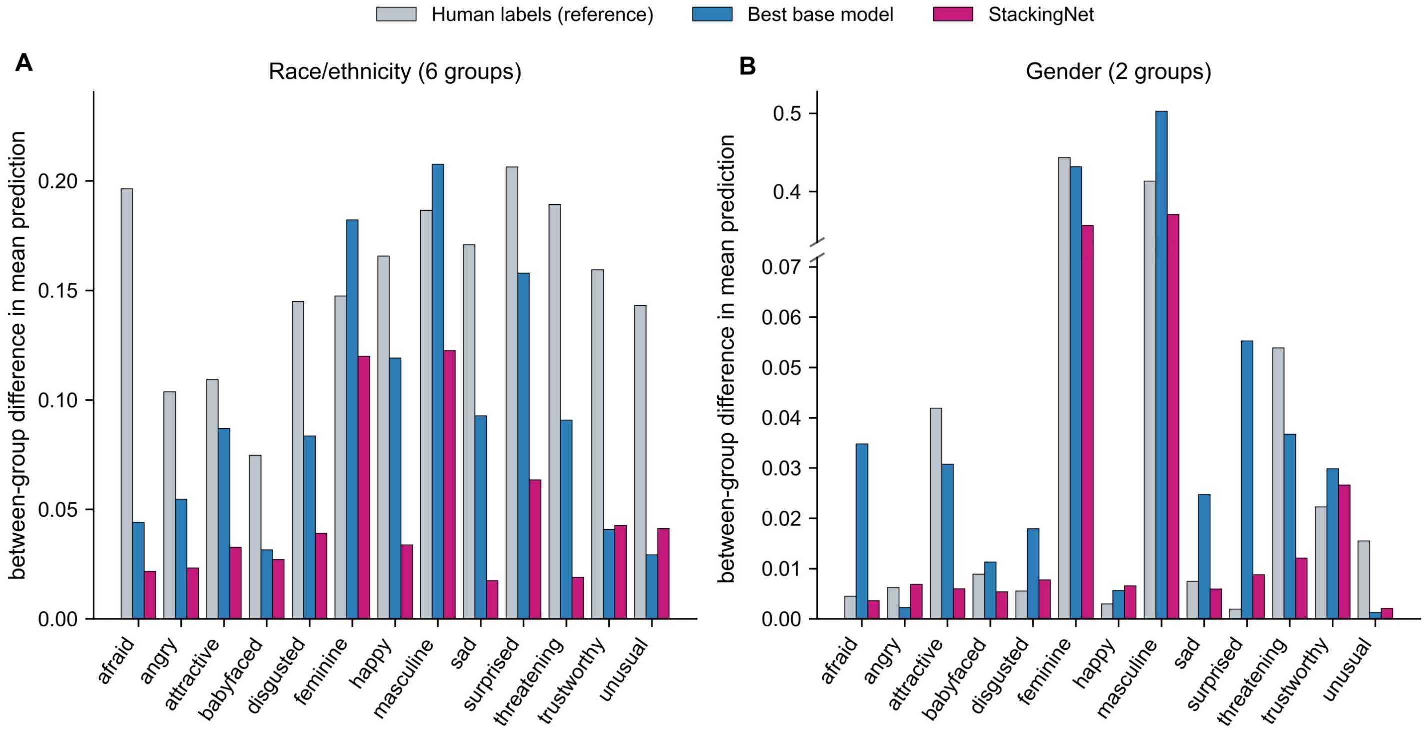

Figure S3: **Demographic-parity view of the Chicago Face Database facial-attribute task. a**, the six race/ethnicity groups. **b**, the two gender groups. Between-group difference in the mean predicted score, the largest minus the smallest per-group mean prediction [1], for the human reference labels, the best single base model, and StackingNet, in the normalized $[0, 1]$ label space. The plotted values are absolute predicted scores rather than errors relative to the human labels, although StackingNet is still trained on the 1% human-annotated labels.

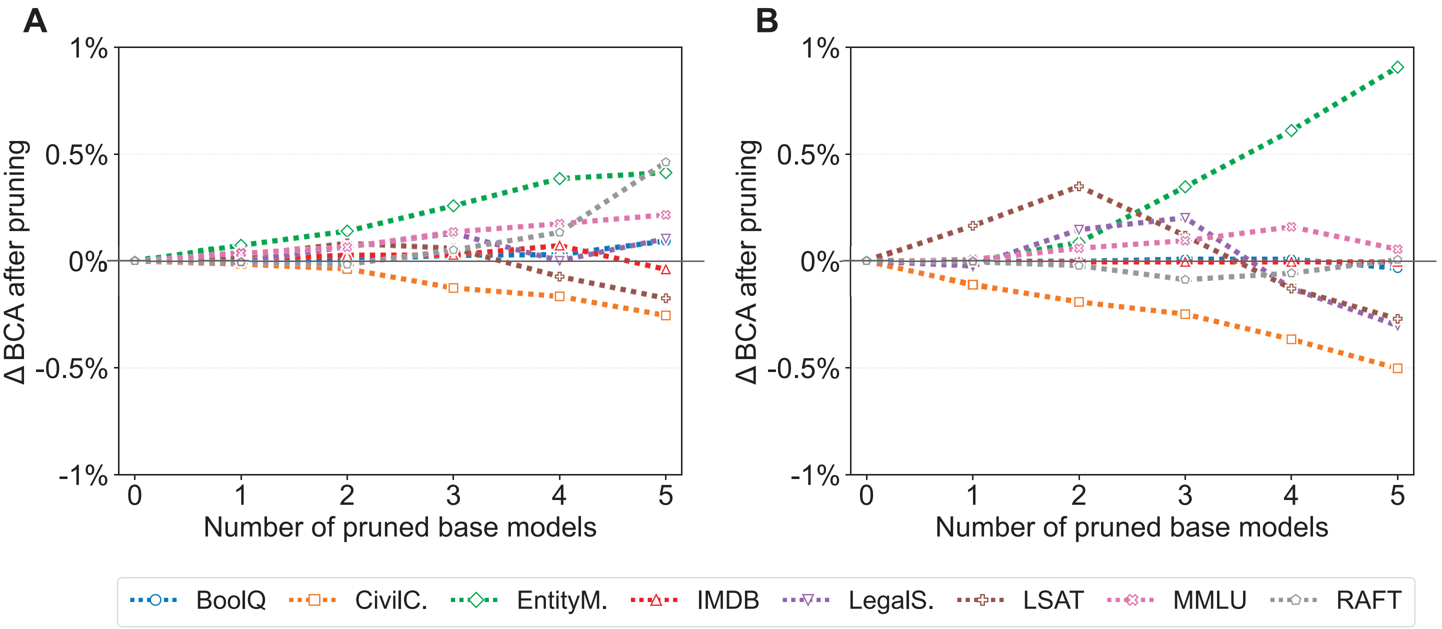

Figure S4: **Performance of StackingNet on classification tasks for adversary pruning.** Relative changes in BCA after sequentially pruning the lowest-weighted base models are shown. **a**, Performance under random-injection attacks. Five randomly predicting base models are injected into the combination. **b**, Performance under label-flipping attacks. Five low-performing base models in the original combination are flipped to predict a different class. Results are averaged over one hundred runs with different random seeds.

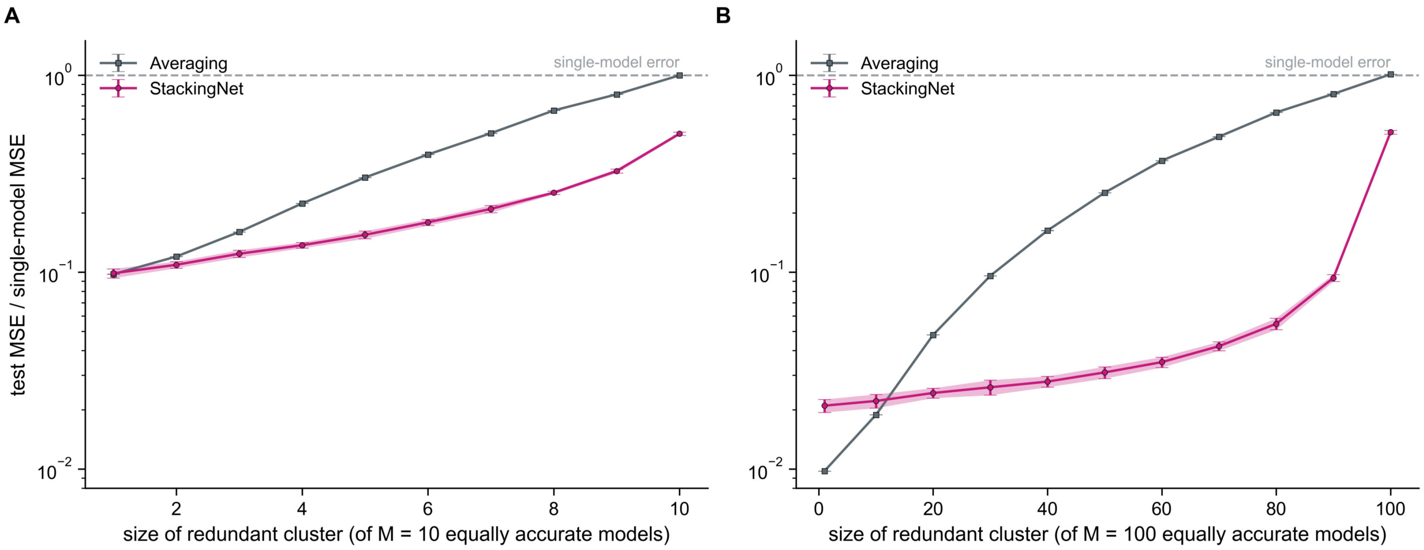

Figure S5: **Test error of averaging and StackingNet as a redundant cluster of base models grows.** Synthetic data: a pool of M regressors of equal error variance, of which a cluster of c models is made mutually redundant by sharing one common error term while the remaining $M - c$ keep independent errors. **a**, $M = 10$. **b**, $M = 100$. The vertical axis is the test mean-squared error relative to the single-model MSE on a logarithmic scale, so the dashed line at one marks the error of any single base model. Each pool draws 10,000 examples, of which 1% are labeled to fit StackingNet. Markers and shaded bands are the mean and standard deviation over 30 repetitions that each resample the labeled subset and reinitialize StackingNet.

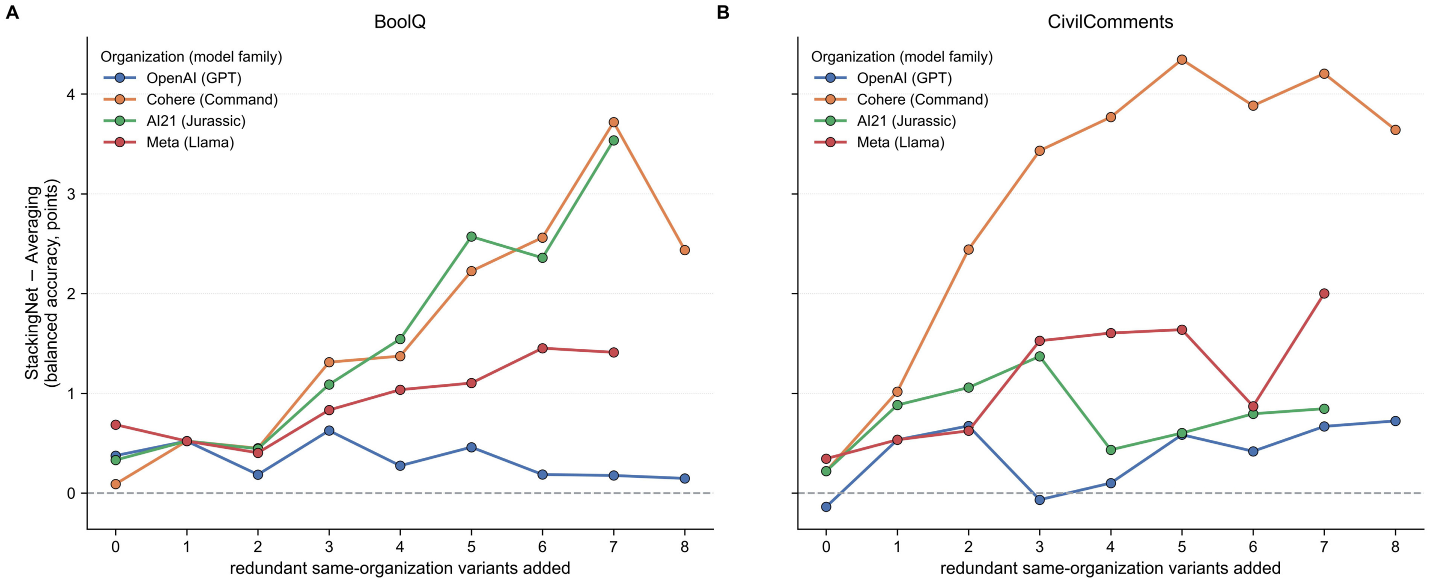

Figure S6: **Effect of adding redundant model variants from a single organization to a diverse pool. a**, BoolQ. **b**, CivilComments. Starting from a fixed pool of five strong models, one from each of five organizations, an increasing number of further variants from one organization is added, that is, models from the same developer that share architecture, training data, and tuning recipe and therefore make correlated errors. Each curve is one such organization. The vertical axis is StackingNet's balanced accuracy minus that of unweighted averaging, in percentage points, against the number of redundant variants added. Both methods use a 5% labeled split, averaged over five seeds, and the dashed line marks equality.

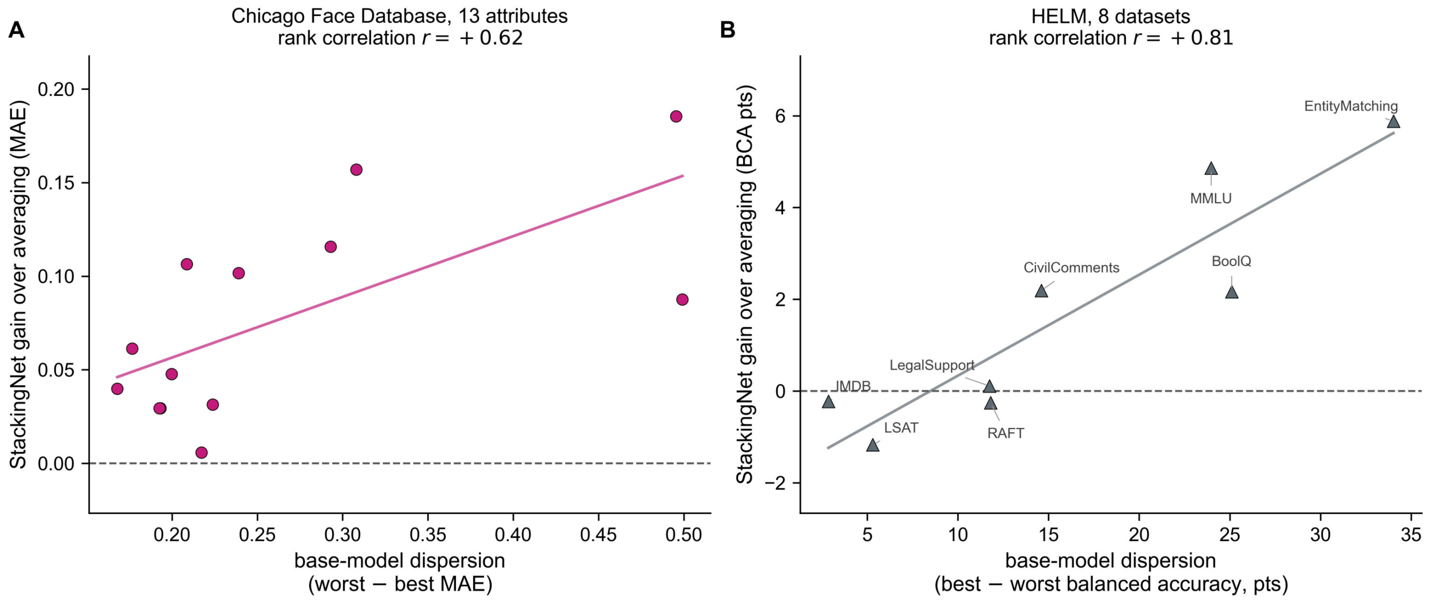

Figure S7: **StackingNet's gain over unweighted averaging versus base-model dispersion. a**, the thirteen Chicago Face Database facial-attribute regression tasks, in MAE. **b**, the eight HELM classification datasets, each labeled by name, in balanced-accuracy points. The horizontal axis is base-model dispersion, the spread between the best and worst base model in the pool. The vertical axis is StackingNet's gain over unweighted averaging of the base models, so a value above the dashed zero line indicates that StackingNet outperforms averaging. In each panel the line is a least-squares fit and the rank correlation coefficient is shown.

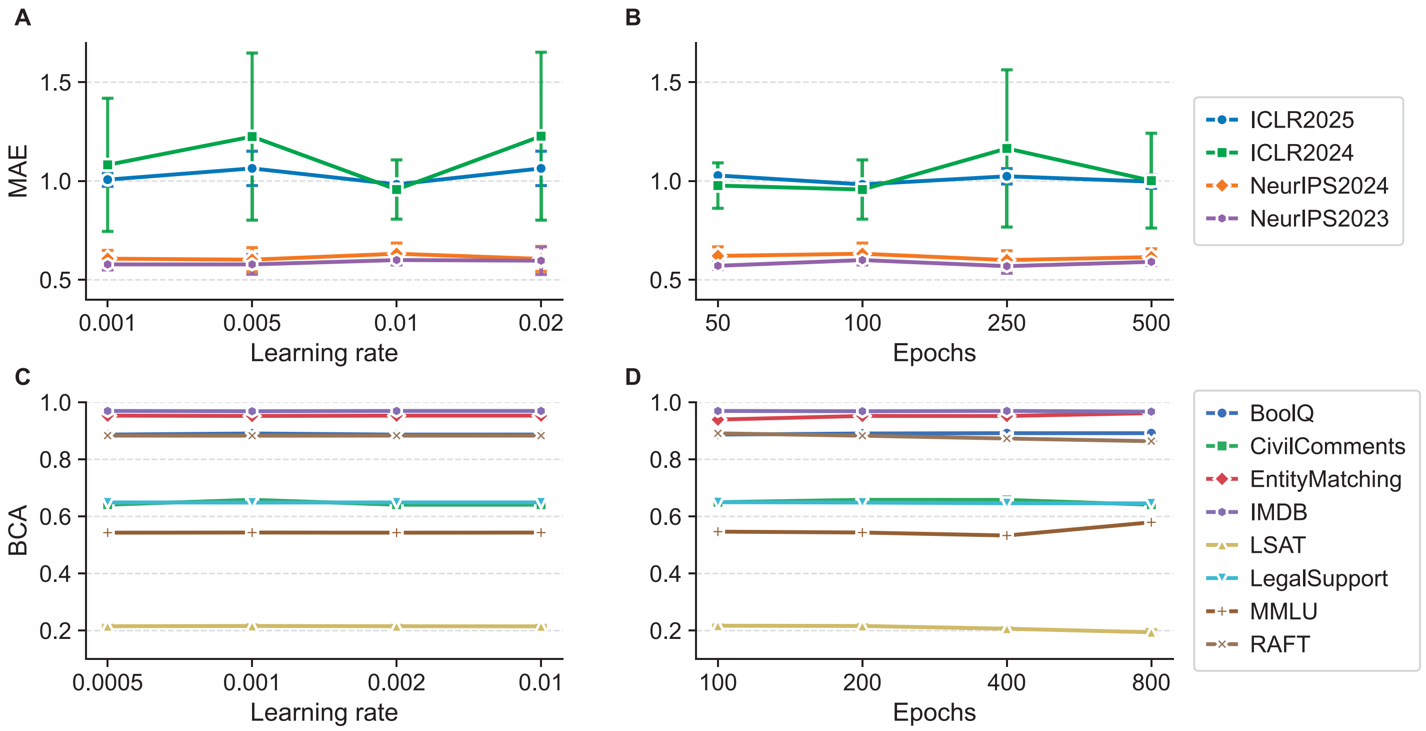

Figure S8: **Sensitivity of StackingNet to different learning rates and training epochs. a-b**, MAE of StackingNet on regression datasets w.r.t. different learning rate and training epochs. **c-d**, BCA of StackingNet on classification datasets w.r.t. different learning rate and training epochs.

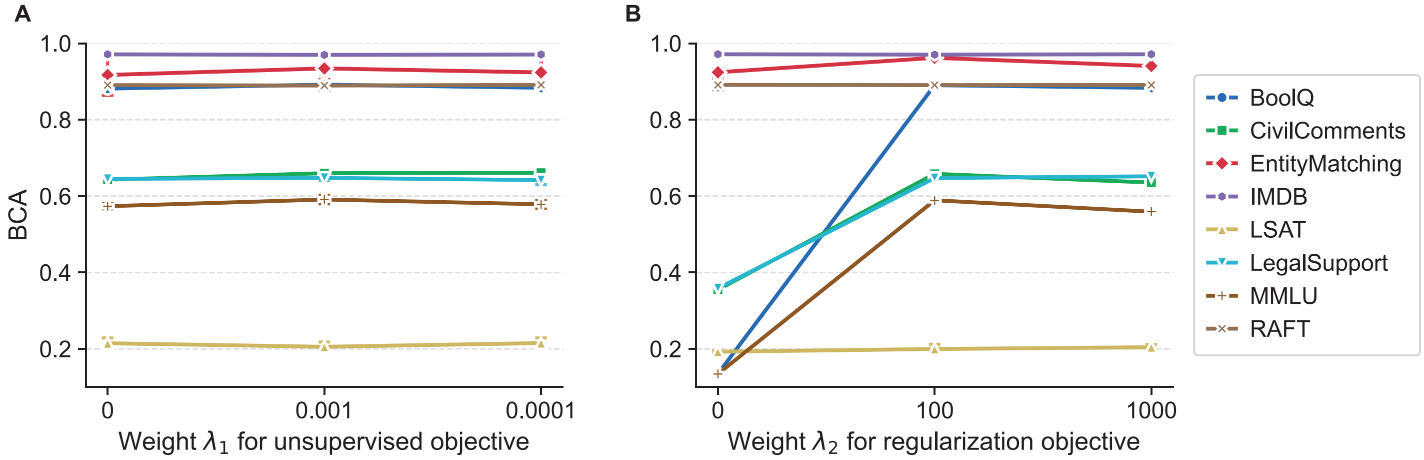

Figure S9: **Sensitivity of StackingNet to different weights for loss integration for classification combination.** **a**, BCA of StackingNet w.r.t. the weight λ_1 for unsupervised loss. **b**, BCA of StackingNet w.r.t. the weight λ_2 for regularization loss. The weight for the supervised objective is fixed at 1.

Table S1: Predicting the peer consensus on paper rating.

Dataset	Single human reviewer		AI predictors		
	Self-inclusive	Held-out	Best single LLM	Averaging	StackingNet
ICLR2025	0.946	1.268	1.248	1.846	**1.123**$_{\pm 0.11}$
ICLR2024	0.938	1.280	1.248	1.857	**1.130**$_{\pm 0.14}$
NeurIPS2024	0.780	1.041	1.092	1.430	**0.789**$_{\pm 0.14}$
NeurIPS2023	0.782	1.016	1.098	1.443	**0.721**$_{\pm 0.10}$

Entries are the mean absolute error against the consensus target, where lower is better.
Self-inclusive: a reviewer scored against a consensus that still includes their own rating.
Held-out: a reviewer scored against the mean rating of the paper's other reviewers only.
The best single LLM, averaging, and StackingNet are all scored against the held-out consensus.
StackingNet is trained few-shot on the held-out reviewer's rating, which is excluded from its scoring target.
Entries are bootstrap means over 50 resamples, and bold marks the lowest held-out error.

Table S2: Inter-model error dependence per task.

Task	Base models	Mean error correlation	Joint-error rate
Regression			
CFD (13 attributes)	10	0.39	–
ICLR2025	6	0.51	–
ICLR2024	6	0.52	–
NeurIPS2024	6	0.37	–
NeurIPS2023	6	0.36	–
Classification			
BoolQ	10	0.35	0.08
CivilComments	10	0.23	0.28
EntityMatching	10	0.14	0.08
IMDB	10	0.34	0.02
LegalSupport	10	0.25	0.23
LSAT	10	0.13	0.64
MMLU	10	0.41	0.36
RAFT	10	0.35	0.07

Mean error correlation: the mean off-diagonal correlation between base-model errors, on the residual (prediction minus label) for regression and the error indicator (one if wrong, zero if right) for classification. The CFD value is averaged over its thirteen attributes.
Joint-error rate: the fraction of items that two base models get wrong together, averaged over all model pairs (the double-fault diversity measure of ref [2]). It is defined only for the classification tasks, so the regression rows are marked "–".

Table S3: Mean absolute error on the CFD dataset for ablation studies.

Dataset	Linear Regression		StackingNet w/o bias		StackingNet w/ bias			
	w/o clamp	weight clamp	w/o clamp	weight clamp	w/o clamp	weight clamp	bias clamp	both clamp
CFD-afraid	1.399	0.512	1.406	0.512	1.406	0.513	1.404	**0.510**
CFD-angry	1.031	0.538	0.974	0.556	0.941	0.526	0.941	**0.524**
CFD-attractive	0.755	0.609	0.764	0.608	0.760	0.608	0.759	**0.606**
CFD-babyfaced	1.032	0.484	0.722	0.484	0.730	0.484	0.728	**0.482**
CFD-disgusted	2.311	0.611	2.115	0.543	2.137	0.611	2.123	**0.508**
CFD-feminine	0.743	0.509	0.736	**0.505**	0.734	**0.505**	0.736	0.507
CFD-happy	1.202	**0.526**	0.822	0.606	0.697	**0.526**	0.695	0.534
CFD-masculine	0.634	0.525	0.631	0.416	0.632	0.520	0.631	**0.408**
CFD-sad	0.832	**0.514**	0.837	0.570	0.830	0.549	0.830	0.548
CFD-surprised	1.235	1.114	1.056	0.591	1.026	1.113	1.016	**0.579**
CFD-threatening	5.743	0.751	1.444	0.540	2.579	0.751	1.443	**0.537**
CFD-trustworthy	3.396	**0.395**	0.792	0.587	0.576	**0.395**	0.571	0.472
CFD-unusual	0.942	0.772	0.855	0.517	0.876	0.768	0.852	**0.455**
Avg.	1.635	0.605	1.012	0.541	1.071	0.979	0.605	**0.513**

Clamp refers to non-negativity constraint.

Table S4: Complete information of the queried LLMs/VLMs.

Model Alias	Complete Version ID	Organization	Parameters	Access / Query Date	URL
DeepSeek-R1	DeepSeek-R1-0528	DeepSeek	671 B	Jul. 17, 2025	DeepSeek
Doubao	doubao-seed-1-6-250615	ByteDance	Not specified	Jul. 7, 2025	ByteDance
Gemini-2	gemini-2.0-flash-001	Google	Not specified	Jul. 15, 2025	GoogleAPIs
GPT-4o	gpt-4o-2024-08-06	OpenAI	Not specified	Jul. 13, 2025	OpenAI
GPT-5	gpt-5-2025-08-07	OpenAI	Not specified	Aug. 11, 2025	OpenAI
Qwen-Turbo	qwen-turbo-2025-04-28	Alibaba Cloud	Not specified	Jul. 18, 2025	AliYun
BLIP	blip-vqa-base	Salesforce	385 M	Jun. 3, 2025	Hugging Face
DeepSeek-VL	deepseek-vl-7b-chat	DeepSeek	7 B	Jun. 3, 2025	Hugging Face
H2OVL	h2ovl-mississippi-2b	H2O.ai	2 B	Jun. 3, 2025	Hugging Face
InternVL-2	InternVL2-8B	OpenGVLab	8 B	Jun. 3, 2025	Hugging Face
LLaVA	llava-onevision-qwen2-7b-si-hf	NTU & ByteDance	7 B	Jun. 3, 2025	Hugging Face
Molmo	Molmo-7B-O-0924	AI2	7 B	Jun. 3, 2025	Hugging Face
Paligemma	paligemma-3b-mix-448	Google	3 B	Jun. 3, 2025	Hugging Face
Phi-3.5	Phi-3.5-vision-instruct	Microsoft	4 B	Jun. 3, 2025	Hugging Face
SAIL-VL	SAIL-VL-1d5-2B	ByteDance	2 B	Jun. 3, 2025	Hugging Face
SmolVLM	SmolVLM-Instruct	Hugging Face	2 B	Jun. 3, 2025	Hugging Face
AnthropicLM-v4	anthropic_stanford-online-all-v4-s3	Anthropic & Stanford	52 B	Nov. 17, 2023	Anthropic
Command	cohere_command-xlarge-beta	Cohere	52.4 B	Nov. 17, 2023	Cohere
Falcon	tiiuae_falcon-40b	Technology Innovation Institute	40 B	Nov. 17, 2023	Hugging Face
GPT-3.5-Turbo	openai_gpt-3.5-turbo-0301	OpenAI	Not specified	Nov. 17, 2023	OpenAI
Jurassic-2	ai21_j2-jumbo	AI21 Labs	178 B	Nov. 17, 2023	AI21
Llama-2	meta_llama-2-70b	Meta	70 B	Nov. 17, 2023	Hugging Face
MPT	mosaicml_mpt-instruct-30b	MosaicML	30 B	Nov. 17, 2023	Hugging Face
Palmyra-X	writer_palmyra-x	Writer	43 B	Nov. 17, 2023	Writer
RedPajama	together_redpajama-incite-instruct-7b	Together AI	7 B	Nov. 17, 2023	Hugging Face
TNLG-v2	microsoft_TNLGv2_530B	Microsoft & Nvidia	530 B	Nov. 17, 2023	Microsoft

LLMs/VLMs are ordered alphabetically.
All queries for the datasets from HELM benchmark were derived from their official release.

Prompts for Research Paper Rating The prompt follows a structured format comprising several components: it assigns the model the role of a reviewer, provides the official review guidelines of the target conference, includes the full manuscript text, and requests both a detailed qualitative assessment and quantitative ratings. The design builds on the structured prompting framework introduced by Yu *et al.* [3]. For the regression task, only the scalar rating is used as supervision. By incorporating these contextual elements, the prompt encourages more grounded and consistent evaluations of complex academic manuscripts, aligning the model's reasoning more closely with the cognitive process of human reviewers. The complete prompt format is shown below.

LLM prompt for research paper rating

[System prompt]
You are an AI researcher reviewing a paper submitted to a prestigious AI research conference. You will be provided with the manuscript text, the conference's reviewer guidelines and templates. Your objective is to thoroughly evaluate the paper, adhering to the provided guidelines and the specified response template. Ensure your evaluation is objective, comprehensive, and aligned with the conference standards.
## Reviewer Guidelines
{Reviewer Guidelines of the Conference}
## Response Template (JSON format)
Provide the review in valid JSON format with the following fields. Ensure all fields are completed as described below. The response must be a valid JSON object.
- "summary_of_the_paper": Briefly summarize the paper and its contributions. This is not the place to critique the paper; the authors should generally agree with a well-written summary. You may use paragraphs and bulleted lists for formatting, but ensure that the content remains a single, continuous text block. Do not use nested JSON or include additional fields.
- "main_review": Provide review comments as a single text field (a string). Consider including assessment on the following dimensions: a comprehensive list of strong and weak points of the paper, your recommendation, supporting arguments for your recommendation, questions to clarify your understanding of the paper or request additional evidence, and additional feedback with the aim to improve the paper. You may use paragraphs and bulleted lists for formatting, but ensure that the content remains a single, continuous text block. Do not use nested JSON or include additional fields.
- "summary_of_the_review": Concise summary of 'main_review'. You may use paragraphs and bulleted lists for formatting, but ensure that the content remains a single, continuous text block. Do not use nested JSON or include additional fields.
- "correctness": A numerical rating on the following scale to indicate that the claims and methods are correct. The value should be between 1 and 4, where:

- *1 = The main claims of the paper are incorrect or not at all supported by theory or empirical results.*
- *2 = Several of the paper's claims are incorrect or not well-supported.*
- *3 = Some of the paper's claims have minor issues. A few statements are not well-supported, or require small changes to be made correct.*
- *4 = All of the claims and statements are well-supported and correct.*

- "technical_novelty_and_significance": A numerical rating on the following scale to indicate technical novelty and significance. The value should be between 1 and 4, where:

- *1 = The contributions are neither significant nor novel.*
- *2 = The contributions are only marginally significant or novel.*
- *3 = The contributions are significant and somewhat new. Aspects of the contributions exist in prior work.*
- *4 = The contributions are significant and do not exist in prior works.*

- "empirical_novelty_and_significance": A numerical rating on the following scale to indicate empirical novelty and significance. The value should be between 1 and 4, or -999 if not applicable, where:

- *1 = The contributions are neither significant nor novel.*
- *2 = The contributions are only marginally significant or novel.*
- *3 = The contributions are significant and somewhat new. Aspects of the contributions exist in prior work.*
- *4 = The contributions are significant and do not exist in prior works.*
- *-999 = Not applicable.*

- "flag_for_ethics_review": A boolean value ('true' or 'false') indicating whether there are ethical concerns in the work.
- "recommendation": A string indicating the final decision, which must strictly be one of the following options: 'strong reject', 'reject, not good enough', 'marginally below the acceptance threshold', 'marginally above the acceptance threshold', 'accept, good paper', or 'strong accept, should be highlighted at the conference'.
- "rating": A float indicating the final decision score in the range [1, 10] that corresponds to the recommendation, where higher scores indicate better paper.
- "confidence": A numerical values to indicate how confident you are in your evaluation. The value should be between 1 and 5, where:

- *1 = You are unable to assess this paper and have alerted the ACs to seek an opinion from different reviewers.*
- *2 = You are willing to defend your assessment, but it is quite likely that you did not understand the central parts of the submission or that you are unfamiliar with some pieces of related work. Math-/other details were not carefully checked.*
- *3 = You are fairly confident in your assessment. It is possible that you did not understand some parts of the submission or that you are unfamiliar with some pieces of related work. Math/other details were not carefully checked.*
- *4 = You are confident in your assessment, but not absolutely certain. It is unlikely, but not impossible, that you did not understand some parts of the submission or that you are unfamiliar with some pieces of related work.*
- *5 = You are absolutely certain about your assessment. You are very familiar with the related work and checked the math/other details carefully.*

[User prompt]
Here is the paper you are asked to review. Write a well-justified review of this paper:
{Full paper text converted from the PDF source}

We used the official OpenReview API (https://github.com/openreview/openreview-py) to retrieve paper PDFs and corresponding human reviewer overall rating scores. To convert to text, the documents were then parsed using the PyMuPDF (https://github.com/pymupdf/PyMuPDF) Python library to extract markdown-style text. To simulate an anonymous peer review setting, we applied regular expression filters to systematically remove identifying details, including author names, institutional affiliations, contact information, funding declarations, publication metadata, etc. The scalar rating values returned by the models were used as regression scores.

Prompts for Facial Image Attribute Rating The prompt is designed to elicit judgments of perceived facial attributes without introducing contextual information, assigned roles, or preference framing. It presents a direct, neutrally worded brief question following the image token input.

VLM prompt for facial image attribute rating

{Image content represented as converted tokens}
On a scale of 1 to 7, how {Adjective} does the person pictured above appear? (1 = Not at all, 4 = Neutral, 7 = Extremely)
Answer in a single float:

Prompts for HELM Datasets Each of the eight datasets in the HELM benchmark provides a predefined textual query. Representative examples are listed in Supporting Information Table S5.

Table S5: Example query prompts from datasets of the HELM benchmark.

Dataset Name	Example
BoolQ	*Passage: The center contact of the bulb typically connects to the medium-power filament, and the ring connects to the low-power filament. Thus, if a 3-way bulb is screwed into a standard light socket that has only a center contact, only the medium-power filament operates. In the case of the 50 W / 100 W / 150 W bulb, putting this bulb in a regular lamp socket will result in it behaving like a normal 100W bulb.*<br>*Question: Do 3 way light bulbs work in any lamp?*<br>*Answer:*
CivilComments	*Passage: she'll be whatever she needs to be in order to gain office. once she's there she'll not give a crap what you think.*<br>*Question: is the passage above toxic?*<br>*Answer:*
EntityMatching	*Are Product A and Product B the same? Yes or No?*<br>*Product A is Beer_Name: Fisherman 's Tea Party. Brew_Factory_Name: Cape Ann Brewing Company. Style: American Barleywine. ABV: 9.00 %.*<br>*Product B is Beer_Name: Cape Ann Fisherman_s Tea Party. Brew_Factory_Name: Cape Ann Brewing Company. Style: Barley Wine. ABV: 9 %.*<br>*Are A and B the same?*<br>*Answer:*
IMDB	*Passage: This film is totally mindblowing. It manages to be thought provoking, funny, tragic, and cinematic yet claustrophobic. Although the flashbacks are unnecessary, the film maintains a pacy, punchy grip and the performances are all excellent, in particular Alec Baldwin, and the mesmerising Eric Bogosian as the film's anti-hero, Barry Champlain.*<br>*Sentiment:*
LegalSupport	*Which statement best supports the passage?*<br>*Passage: A truly vindictive prosecution is illegal because it violates due process.*<br>*A. federal prosecution may proceed if federal prosecutor did not participate in state prosecutor's allegedly vindictive action against defendant.*<br>*B. vindictiveness may play no part in resentencing of criminal defendant who has successfully invoked right to appeal.*<br>*Answer:*
LSAT	*The following are multiple choice questions. (with answers)*<br>*Bird-watchers explore a forest to see which of the following six kinds of birds-grosbeak, harrier, jay, martin, shrike, wren-it contains. The findings are consistent with the following conditions: If harriers are in the forest, then grosbeaks are not. If jays, martins, or both are in the forest, then so are harriers. If wrens are in the forest, then so are grosbeaks. If jays are not in the forest, then shrikes are.*<br>*Question: Which one of the following is the maximum number of the six kinds of birds the forest could contain? A. two B. three C. four D. five E. six*<br>*Answer:*
MMLU	*The following are multiple choice questions (with answers) about computer security.*<br>*Question: The ____________ is anything which your search engine cannot search.*<br>*A. Haunted web B. World Wide Web C. Surface web D. Deep Web*<br>*Answer:*
RAFT	*Label whether the following tweet contains hate speech against either immigrants or women. Hate Speech (HS) is commonly defined as any communication that disparages a person or a group on the basis of some characteristic such as race, color, ethnicity, gender, sexual orientation, nationality, religion, or other characteristics.*<br>*Possible labels: 1. hate speech 2. not hate speech*<br>*Tweet: It's clear dat the administration's attempts to punish local governments fa refusing to cooperate wit immigration enforcement r unconstitutional. Da administration should focus on treating immigrants w/ compassion and respect.*<br>*Label:*

Each full query includes few-shot exemplars with answers.
For clarity and brevity, only the final query prompt of a single sample is shown.

References

1. E. Chzhen, C. Denis, M. Hebiri, L. Oneto, and M. Pontil, "Fair Regression with Wasserstein Barycenters," in *Advances in Neural Information Processing Systems* (NeurIPS, 2020), 7321–7331.

2. L. I. Kuncheva and C. J. Whitaker, "Measures of Diversity in Classifier Ensembles and Their Relationship with the Ensemble Accuracy," *Machine Learning* 51, no. 2 (2003): 181–207, https://doi.org/10.1023/A:1022859003006.

3. S. Yu, M. Luo, A. Madusu, V. Lal, and P. Howard, "Is Your Paper Being Reviewed by an LLM? Benchmarking AI Text Detection in Peer Review," preprint, arXiv, 2025, https://doi.org/10.48550/arXiv.2502.19614.
